# Clinical characteristics and quality of life in seborrheic dermatitis patients: a cross-sectional study in China

**DOI:** 10.1186/s12955-020-01558-y

**Published:** 2020-09-16

**Authors:** Meiling Xuan, Chuanjian Lu, Zehui He

**Affiliations:** 1 Key Unit of Methodology in Clinical Research, The Second Affiliated Hospital of Guangzhou University of Chinese Medicine, Guangzhou, Guangdong China; 2 State Key Laboratory of Dampness Syndrome of Chinese Medicine, The Second Affiliated Hospital of Guangzhou University of Chinese Medicine, Guangzhou, Guangdong China; 3 Department of Dermatology, The Second Affiliated Hospital of Guangzhou University of Chinese Medicine, Guangzhou, Guangdong China; 4 Guangdong Provincial Key Laboratory of Clinical Research on Traditional Chinese Medicine Syndrome, The Second Affiliated Hospital of Guangzhou University of Chinese Medicine, Guangzhou, Guangdong China; 5 Department of Clinical Epidemiology, The Second Affiliated Hospital of Guangzhou University of Chinese Medicine, No.111 Da De Road, Yue Xiu District, Guangzhou, 510120 Guangdong China

**Keywords:** Seborrheic dermatitis, Skindex-29, Quality of life

## Abstract

**Background:**

Seborrheic dermatitis (SD) is a common, chronic, inflammatory skin disorder, yet few studies have reported its clinical characteristics, or addressed its effect on quality of life (QoL). This study assesses the clinical characteristics and QoL of SD patients in China. It also identifies the clinical, demographic and environmental factors that may influence QoL.

**Methods:**

Three hundred twelve SD outpatients from 9 hospitals completed a survey. QoL was measured with the dermatology-specific instrument Skindex-29. We collected social demographic characteristics and disease severity, and conducted logistic regression to determine the factors associated with QoL impairment.

**Results:**

67.3% of the patients were females. The mean Skindex-29 overall score was 33.97 (SD = 20.55). The breakdown was 40.79 (SD = 24.24) for emotions, 32.83 (SD = 19.84) for symptoms and 28.3 (SD = 23.24) for functioning. 48.1% had severe emotional problems. Logistic regression analysis showed that BMI less than 25 (OR = 0.223; 95% CI: 0.072–0.692; *P* = 0.009), skin disease-related hospitalization (OR = 6.882; 95% CI:1.767–26.795; *P* = 0.005), environmental PM 10 levels above 120 μg/m^3^ (OR = 3.386; 95% CI: 1.253–9.15; *P* = 0.016) and severe disease conditions (OR = 4.438; 95% CI:1.26–15.626; *P* = 0.02) were risk factors for severe emotional impairment. Moreover, skin disease-related hospitalization (OR = 6.057; 95% CI:1.351–27.149; *P* = 0.019), environmental PM 10 levels between 70 and 120 μg/m^3^ (OR = 6.317; 94% CI: 1.704–23.42; *P* = 0.006), moderate (OR = 2.388; 95% CI: 1.272–4.487; *P* = 0.007) and severe disease conditions (OR = 5.732; 95% CI: 1.838–17.88; *P* = 0.003) were each risk factors for overall severe impairment.

**Conclusion:**

In China, nearly half of SD patients had severely emotional problems. Disease severity, BMI, dermatologic hospitalization, and ambient PM 10 levels are each risk factors for QoL impairment in SD patients. These implications are alarming, and warrant public health attention in SD disease management.

## Background

Seborrheic dermatitis (SD) is a chronic, superficial, inflammatory skin disorder, characterized by scaling on an erythematous base [1]. It is considered one of the most frequent dermatoses. The etiology of SD is complex, and may be associated with several factors or internal diseases [2]. It also has high incidence and prevalence (1 to 3% in the immunocompetent adult population, 3 to 5% in young adults, and 40 to 80% in HIV-positive individuals) [3, 4] An Asian survey has shown that the prevalence of SD was 2.66, 2.85, 17.16 and 26.45% in Macao, Guangzhou, Malaysia and Indonesia, respectively [5]. The actual prevalence is probably much higher, and men are affected more frequently than women.

Patients with SD have scaling scalps, and erythematous patches on the eyebrows, eyelids, nasolabial creases, lips, ears and sternal area. These symptoms affect patients’ social activities. SD co-occurs with depression, anxiety and other emotional symptoms, and has serious passive effects on patients’ Quality of Life (QoL). However, even though it is common and socially embarrassing, few studies have assessed SD patients’ QoL. SF-36, Dermatology Life Questionnaire Index (DLQI) and Skindex 29 have been used worldwide to investigate QoL [6–9]. Yet there is no data in systematic review studies concerning the effects of topical anti-inflammatory therapies on SD patients’ quality of life [10]. Some clinical trials of SD have reported QoL as a secondary outcome measure by using DLQI and Scalpdex [11, 12]. Likewise, a handful of studies have focused on QoL among patients suffering from this skin disorder in China.

Recent evidence has indicated that particulate matter (PM) causes cutaneous damage not only directly, but also indirectly [13]. Systematic review has confirmed that particulate matter (PM) (PM 10 and PM 2.5) are associated with increased risks of human skin diseases [14]. It has also been suggested that other pollutants, such as O_3,_ exert indirect toxic mechanisms on the skin [15, 16]. Whereas, there is lack of evidence about the effect of air pollution on QoL among SD patients in Asia. Therefore, this study investigates the clinical characteristics and QoL of patients with SD in China, and explores factors associated with QoL impairment.

## Methods

### Study design

We conducted a cross-sectional survey involving Chinese adults with seborrheic dermatitis. The inclusion criteria were: at least 16 years of age; diagnosed with SD; provided signed informed consent.

### Settings

This study was conducted in 9 general hospitals, in 6 Chinese cities, from 2013 to 2015. Four of the hospitals were located in subtropical Southern China. The other 5 hospitals were located in temperate climate zones. All participants gave signed informed consent. This study was approved by the ethics committee at the Guangdong Provincial Hospital of Chinese Medicine.

### Sociodemographic characteristics

Patients were asked to provide their sociodemographic information such as age, sex, disease duration, body mass index (BMI), relationship status, education level, employment, smoking and alcohol consumption, exercise habits, hospitalizations and private insurance status.

### Health-related characteristics

We assessed this group of patients’ QoL with Skindex-29. Skindex-29 is one of the best dermatological instruments for measuring dermatology-specific QoL. It is a 30-item dermatology-specific QoL instrument for adults with an unscored item no. 18, measuring 3 domains—emotions, functioning and symptoms. Each item is rated on a 5-point Likert scale (never, rarely, sometimes, often, all the time), with higher scores indicating worse health status. There is also a Chinese version of the Skindex-29. It has been shown to be reliable and valid for use as a QoL instrument for patients with skin disease in China [17].

In this study, data were collected during clinical visits for both outpatients and inpatients. After obtaining informed consent, experienced doctors rated SD severity with three grades (mild, moderate, severe). Then patients were asked to complete a survey questionnaire comprised of sociodemographic characteristics and Skindex-29 questions.

### Urban air quality data

Nine hospitals participated in this study. They were located in Guangzhou, Beijing, Shanghai, Chengdu, Urumqi and Harbin. The environmental conditions from 2013 to 2015 (including annual average SO_2_, NO_2_, CO, PM2.5 and PM 10 levels) were gathered from China Statistical Yearbook [18–20]. The average environmental factors from 2013 to 2015 were as follows: SO_2_ 29.8 ± 15.4 μg/m^3^; NO_2_ 52.4 ± 4.3 μg/m^3^; CO 2.2 ± 1.2 μg/m^3^; O_3_ 136.9 ± 36.2 μg/m^3^; PM 2.5 65.3 ± 14.9 μg/m^3^ and PM 10 99.8 ± 29.3 μg/m^3^. Environmental indexes for these six cities are shown in Additional file 1.

### Sampling

This study employed convenience sampling, and patients visiting hospitals were recruited to participate in the survey study when they had satisfied the inclusion criteria. The sample size was determined according to a rule of thumb that the sample size must be 5–10 times the number of survey items. Since there were 30 items, we enrolled 300 patients in this study to satisfy the sample size estimation.

### Statistical analysis

Data were statistically analyzed with PASW Statistics 18.0 (IBM SPSS Inc., Armonk, NY, USA). Patients’ sociodemographic characteristics are shown with descriptive statistics. Mean values and standard deviations were calculated for all QoL scores and compared between subgroups using a *t*-test, nonparametric test or ANOVA. Variables with *P* values ≤0.1 were incorporated into the logistic regression model. We conducted logistic regression analysis with dichotomized Skindex-29 (emotions, symptoms, functioning and overall) mild and moderate (coded 0) and severe (coded 1) as dependent variables, and demographic and disease-related variables as covariates. Severely impaired QoL was based on the Skindex-29 cut-off scores as follows: ≥44 on the overall score, ≥39 on emotions, ≥52 on symptoms and ≥ 37 on functioning [21]. Variables entered the models via the forward likelihood ratio method. *P* < 0.05 was considered statistically significant.

## Results

### Patient characteristics

67.3% of the 312 patients were females, and the mean age was 30.51 years (standard deviation SD = 9.77). Disease duration ranged from 0.02 to 20.5 years (mean = 2.51 years; SD = 3.44). Two hundred seventy-one patients (86%) had normal weight, i.e., BMI was under 25. 26% had been hospitalized for various skin conditions over the past year (Table 1).

**Table 1:** Patient demographic characteristics, urban air quality and disease conditions

	Total, *n* (%)	Missing, *n* (%)
All patients	312 (100)	
Sex		1 (0.3)
Male	101 (32.4)	
Female	210 (67.3)	
Age		0
< 24 years	102 (32.7)	
≥ 24 years	210 (67.3)	
Duration		24 (7.7)
< 3 years	209 (67.0)	
≥ 3 years	79 (25.3)	
BMI^a^		10 (3.2)
< 25	271 (86.0)	
≥ 25	31 (9.8)	
Relationship status		9 (2.9)
Married	153 (49.0)	
Single	150 (48.1)	
Highest level of education		4 (1.3)
High school education or less	103 (3.3)	
College or above	205 (65.7)	
Employment		6 (1.9)
Employed	175 (56.1)	
Unemployed/student	131 (42.0)	
Diet preference		136 (43.6)
No	59 (18.9)	
Yes	117 (37.5)	
Smoking		5 (1.6)
No	197 (63.1)	
Yes	110 (35.3)	
Alcohol consumption		4 (1.3)
No	174 (55.8)	
Yes	134 (42.9)	
Exercise		7 (2.2)
No	134 (42.9)	
Yes	171 (54.8)	
Hospitalized for skin disease over the previous year		7 (2.2)
No	224 (71.8)	
Yes	81 (26.0)	
Income		
≤ 4000 yuan per month	200 (64.1)	
> 4000 yuan per month	112 (35.9)	
Medical insurance		24 (7.7)
No	80 (25.6)	
Yes	213 (68.3)	
SO_2_ level in the air		
< 20 μg/m^3^	133 (42.6)	
20–40 μg/m^3^	85 (27.2)	
> 40 μg/m^3^	94 (30.1)	
NO_2_ level in the air		
≤50 μg/m^3^	123 (39.4)	
> 50 μg/m^3^	189 (60.6)	
CO level in the air		
< 2 μg/m^3^	199 (63.8)	
2–4 μg/m^3^	93 (29.8)	
> 4 μg/m^3^	20 (6.4)	
O_3_ level in the air		
< 100 μg/m^3^	38 (12.2)	
100–160 μg/m^3^	131 (42.0)	
> 160 μg/m^3^	143 (45.8)	
PM 2.5 level in the air		
< 60 μg/m^3^	128 (41.0)	
60–80 μg/m^3^	159 (51.0)	
> 80 μg/m^3^	25 (8.0)	
PM 10 level in the air		
< 70 μg/m^3^	112 (35.9)	
70–120 μg/m^3^	141 (45.2)	
> 120 μg/m^3^	59 (18.9)	
Disease severity		9 (2.9)
Mild	144 (46.2)	
Moderate	138 (44.2)	
Severe	21 (6.7)	

^a^ The BMI classification is based on the World Health Organization’s obesity criteria

### Skindex-29 scores across subgroups

All SD patients were divided into subgroups according to sex, age, disease duration, BMI, marital status, education level, employment, diet preference, smoking, alcohol consumption, routine exercise, hospitalization for skin problems, monthly income, medical insurance, urban air quality levels and disease severity. A comparison of these groups’ Skindex-29 scores showed a statistically significant difference between variable categories, including BMI (*Z* = − 2.418, *P* = 0.016), medical insurance (*Z* = − 2.146, *P* = 0.032), disease severity (*F* = 10.349, *P* < 0.001) in the emotion domain; sex (*Z* = − 2.477, *P* = 0.013) and disease severity (*F* = 13.785, *P* < 0.001) in the symptom domain; BMI (*Z* = − 2.515, *P* = 0.012), hospitalization (*Z* = − 2.745, *P* = 0.006), medical insurance (*Z* = − 2.147, *P* = 0.032), O_3_ levels (*F* = 6.47, *P* = 0.002), PM 2.5 levels (*F* = 7.1, *P* = 0.001), PM 10 levels (*F* = 3.387, *P* = 0.035) and disease severity (*F* = 18.049, *P* < 0.001) in the functioning domain; BMI (*Z* = − 2.277, *P* = 0.023), hospitalization (*Z* = − 2.432, *P* = 0.015) and disease severity (*F* = 16.531, *P* < 0.001) in the overall score (Table 2).

**Table 2:** Comparison of Skindex-29 domains across subgroups (mean ± SD)

	Emotions	Symptoms	Functioning	Overall
Mean	40.79 ± 24.24	32.83 ± 19.84	28.3 ± 23.24	33.97 ± 20.55
Sex				
Male	38.71 ± 25.07	28.72 ± 19.17^*^	27.57 ± 23.95	31.56 ± 20.74
Female	41.8 ± 23.9	34.75 ± 19.95^*^	28.66 ± 22.99	35.12 ± 20.46
Age				
< 24	44.78 ± 25.53	31.05 ± 19.42	29.66 ± 24.02	35.08 ± 20.8
≥ 24	38.82 ± 23.39	33.71 ± 20.03	27.63 ± 22.87	33.42 ± 20.46
Duration				
< 3 year	39.24 ± 24.52	32.13 ± 19.81	26.9 ± 23.48	32.79 ± 20.54
≥ 3 years	44.32 ± 24.12	35.02 ± 20.68	31.41 ± 23.31	36.95 ± 21.19
BMI				
< 25	41.72 ± 24.23^*^	33.01 ± 20.18	29.17 ± 23.49^*^	34.67 ± 20.74^*^
≥ 25	31.38 ± 24.95^*^	28.83 ± 17.95	19.32 ± 20.99^*^	25.94 ± 19.28^*^
Marital status				
Married	38.44 ± 23	33.9 ± 20.8	27.56 ± 22.86	33.27 ± 20.42
Single	42.87 ± 25.23	31.64 ± 19.03	28.77 ± 23.72	34.43 ± 20.76
Education level				
Primary school or high school	40.32 ± 24.36	33.13 ± 17.67	28.84 ± 22.99	34.01 ± 20.42
University	40.97 ± 24.26	32.71 ± 20.88	28.1 ± 23.48	33.97 ± 20.7
Occupation				
Employed	39.43 ± 22.86	32.08 ± 19.38	28.16 ± 22	33.2 ± 19.84
Unemployed/student	42.52 ± 25.89	33.85 ± 20.53	28.48 ± 24.94	34.95 ± 21.51
Taste preferences				
No	47.29 ± 27.42	33.87 ± 19.26	33.9 ± 24.89	38.25 ± 22.23
Yes	41.04 ± 22.73	31.86 ± 20.29	28.84 ± 23.93	33.92 ± 20.15
Smoking				
No	39.12 ± 23.86	32.22 ± 19.76	26.47 ± 21.82	32.58 ± 19.81
Yes	43.58 ± 25.01	33.72 ± 20.29	31.75 ± 25.48	36.35 ± 21.92
Alcohol consumption				
No	39.51 ± 23.89	32.21 ± 19.66	26.88 ± 22.17	32.84 ± 19.83
Yes	42.27 ± 24.82	33.52 ± 20.28	30.33 ± 24.57	35.39 ± 21.58
Exercises				
No	39.66 ± 25.21	32.36 ± 20.01	25.93 ± 22.73	32.65 ± 21.05
Yes	41.16 ± 23.67	32.89 ± 19.83	29.85 ± 23.79	34.68 ± 20.31
Skin disease hospitalization				
No	38.25 ± 23.33	32.01 ± 18.99	25.63 ± 21.08^*^	31.99 ± 19.13^*^
Yes	47.62 ± 25.62	35.45 ± 21.48	35.24 ± 26.91^*^	39.37 ± 23.02^*^
Income				
≤ 4000 yuan/month	41.32 ± 24.43	33.32 ± 20.18	27.55 ± 22.4	34.07 ± 20.21
>4000 yuan/month	39.86 ± 23.99	31.95 ± 19.27	29.64 ± 24.7	33.8 ± 21.25
Medical insurance				
No	46 ± 25.22^*^	37.46 ± 23.02	34.01 ± 26.83^*^	39.16 ± 23.18^*^
Yes	39.07 ± 23.99^*^	31.41 ± 18.24	26.29 ± 21.69^*^	32.29 ± 19.35^*^
Disease severity				
Slight or mild	35.98 ± 22.19^*^	29.1 ± 17.66^*^	23.92 ± 19.25^*^	29.67 ± 17.67^*^
Moderate	43.15 ± 24.6^*^	33.86 ± 20.36^*^	29.18 ± 23.14^*^	35.41 ± 20.7^*^
Severe	59.76 ± 27.46^*^	52.38 ± 21.62^*^	54.96 ± 32.64^*^	55.7 ± 25.78^*^
Urban air quality (SO_2_)				
< 20 μg/m^3^	42.58 ± 21.96	31.71 ± 18.7	30.69 ± 21.86	34.99 ± 19.01
20–40 μg/m^3^	38.72 ± 27.06	33.25 ± 20.55	28.73 ± 26.17	33.44 ± 22.31
> 40 μg/m^3^	40.08 ± 24.82	34.08 ± 20.91	24.48 ± 22.13	32.96 ± 21.25
Urban air quality (NO_2_)				
≤ 50 μg/m^3^	42.78 ± 22.34	31.16 ± 18.78	30.62 ± 22.19	34.85 ± 19.3
> 50 μg/m^3^	39.47 ± 25.4	33.96 ± 20.5	26.76 ± 23.84	33.38 ± 21.39
Urban air quality (CO)				
< 2 μg/m^3^	41.63 ± 22.51	32.34 ± 20.04	28.87 ± 22.68	34.32 ± 19.95
2–4 μg/m^3^	39.56 ± 27.8	34.22 ± 19.61	27.31 ± 24.48	33.71 ± 21.9
> 4 μg/m^3^	38.03 ± 24.32	31.15 ± 19.55	27.08 ± 24.00	31.47 ± 21.05
Urban air quality (O_3_)				
< 100 μg/m^3^	41.64 ± 25.1	32.71 ± 18.54	25.56 ± 20.21^*^	33.33 ± 19.97
100–160 μg/m^3^	36.78 ± 23.73	33.41 ± 20.54	23.58 ± 22.39^*^	31.2 ± 20.34
> 160 μg/m^3^	44.24 ± 24.1	32.35 ± 19.66	33.35 ± 23.84^*^	36.64 ± 20.69
Urban air quality (PM 2.5)				
< 60 μg/m^3^	42.62 ± 22.52	31.94 ± 19.26	31.94 ± 22.79^*^	35.5 ± 19.87
60–80 μg/m^3^	38.66 ± 24.3	33.39 ± 19.92	23.18 ± 21.21^*^	31.78 ± 19.93
> 80 μg/m^3^	42.8 ± 28.66	33.57 ± 21.68	35.26 ± 27.64^*^	37.04 ± 24.23
Urban air quality (PM 10)				
< 70 μg/m^3^	43.1 ± 23.02	31.44 ± 19.01	31.75 ± 22.74^*^	35.43 ± 19.93
70–120 μg/m^3^	41.88 ± 24.73	34.05 ± 21.03	28.13 ± 23.92^*^	34.76 ± 21.4
> 120 μg/m^3^	33.75 ± 24.49	32.64 ± 18.6	22.05 ± 21.52^*^	29.23 ± 19.31

One-way ANOVA/*t*-test/nonparametric described the mean Skindex-29 for various demographics and clinical and environmental variables

*P < 0.05

The comparison of whether or not there was severe impairment, defined by Skindex-29 cutoff scores for the subgroups, are shown in Additional file 2. 130 (42.2%), 75 (24.4%), 66 (21.4%) and 86 (27.9%) patients had severe impairment on emotion, symptoms, functioning and the overall realm, respectively. The high Skindex-29 scores in each of the domains was significantly different in terms of SD severity (emotion: *P* = 0.001; symptoms: *P* < 0.001; functioning: *P* < 0.001; overall: *P* < 0.001). Emotion and overall scores differed for hospitalization (*P* = 0.011; *P* = 0.002) and medical insurance (*P* = 0.016; *P* = 0.048) subgroups. The emotion score was also significantly different for several variables, including patient’s BMI and the concentration of PM 10 in the air (*P* = 0.014; *P* = 0.025). Also, the functional impact differed on O_3_ concentration in the air (*P* = 0.035). Moreover, those severely impaired based on their overall score also varied on smoking and alcohol consumption (*P* = 0.023; *P* = 0.034) subgroups.

### Skindex-29 impairment risk factors

Variables with *P* value < 0.1 in Additional file 2 were included in a multivariate logistic regression model (emotions: duration, BMI, smoking, hospitalization, medical insurance, PM 10 level and disease severity; symptoms: disease severity; functioning: diet preference, smoking, hospitalization, PM 2.5 levels, O_3_ levels and disease severity; overall: BMI, smoking, alcohol consumption, hospitalization, medical insurance, O_3_ level, PM 10 level and disease severity). They were selected by the forward likelihood ratio method in the model. Significant variables for the multiple logistic model are shown in Table 3.

**Table 3:** Logistic regression analysis with Skindex scores as the dependent variable

Variable	Emotion		Symptoms		Functioning		Overall	
	Exp(B)	95% CI	Exp(B)	95% CI	Exp(B)	95% CI	Exp(B)	95% CI
BMI								
< 25	Ref							
≥ 25	0.223	0.072–0.692						
Skin disease-related hospitalization								
No	Ref						Ref	
Yes	6.882	1.767–26.795					6.057	1.351–27.149
PM 10 level in the air								
< 70 μg/m^3^	Ref						Ref	
70–120 μg/m^3^	0.898	0.202–3.995					6.317	1.704–23.42
> 120 μg/m^3^	3.386	1.253–9.15					3.742	0.674–20.77
Disease severity								
Mild	Ref		Ref		Ref		Ref	
Moderate	1.686	0.953–2.982	1.872	1.049–3.341	2.01	0.844–4.789	2.388	1.272–4.487
Severe	4.438	1.26–15.626	8.057	3.012–21.552	10.885	2.676–44.277	5.732	1.838–17.88

*Ref* indicates reference variable

BMI less than 25, skin disease-related hospitalization, environmental PM 10 concentration greater than 120 μg/m^3^ and disease severity of moderate to severe were risk factors for severe emotional impairment (*P* = 0.009*, P* = 0.005*, P* = 0.04*, P* = 0.03). The odds ratios were 0.223 (< 25 versus ≥25), 6.882 (yes versus no), 3.386 (> 120 μg/m^3^ versus < 70 μg/m^3^), 1.686 and 4.438 (moderate versus mild, severe versus mild) respectively. Disease severity for moderate to severe was the only factor associated with symptoms and severely impaired functioning (*P* < 0.01). The odds ratios were 1.872 and 8.057 (moderate versus mild, severe versus mild) in the symptom domain, 2.01 and 10.885 (moderate versus mild, severe versus mild) in the function domain. Moreover, skin disease-related hospitalization, environmental PM 10 concentration around 70–120 μg/m^3^ and disease severity of moderate to severe were risk factors for overall severe impairment (*P* = 0.019*, P* = 0.011*, P* = 0.006). The odds ratios were 6.057 (yes versus no), 6.317 (70–120 μg/m^3^ versus < 70 μg/m^3^), 2.388 and 5.732 (moderate versus mild, severe versus mild), respectively.

## Discussion

This study has demonstrated that the presence of SD has a negative effect on QoL, and that disease severity influences QoL in all domains, and in the overall score. This study’s sample size was larger than previous studies conducted in Asia [8, 22]. In addition to disease severity, hospitalization and BMI, the environment was also found to influence QoL.

Individuals were most influenced in the emotion domain. Also, skin disease-related hospitalization influenced individual feelings, which incurs substantial costs for both patients and the healthcare system. Obese or overweight people suffered less from emotional problems. This may have been because they pay less attention to appearance than those who stay in shape. In addition, dermatologic hospitalization and PM 10 concentration also negatively affected overall QoL scores.

Several instruments have been used to evaluate SD patients’ QoL, such as the SF-36, DLQI and Skindex-29. Dai et al. used the SF-36 to assess QoL impairment in Chinese patients, which revealed that QoL was poor and was related to depression, alcohol consumption, smoking, exercise, and spicy food consumption [6]. We also demonstrated that alcohol consumption and smoking influenced patients’ QoL. A previous study found that SD patients’ mean DLQI score was 7.73, and that female, younger patients with higher education levels were the independent factors influencing QoL in Poland [7]. On the contrary, neither sex, age, nor education levels were independent QoL factors in this study. Furthermore, our data indicated that Chinese patients have a lower QoL than patients in other countries. A Korean study of soldiers with seborrheic dermatitis reported lower Skindex-29 scores for emotional and functioning than the scores in our study (E: 27.8 vs. 40.79; F: 19.6 vs. 28.29) [8]. This may have been due to disparities between military and common people in terms of social environment and physical health conditions. Compared to patients in Spain, patients in this study had worse QoL along all three Skindex domains (E: 20.54, S: 30.14 vs. 32.91, F: 15.45 vs. 28.29) [9]. Unlike previous studies, we analyzed the factors associated with the Skindex-29 cut-off score. This provides a better definition for the severe impairment in each domain.

We found a statistically significant correlation between ambient PM 10 levels and patients’ QoL in our study. When outdoor air quality worsens (PM 10 levels over 120 μg/m^3^), individuals suffer more emotional torture than ever (OR:3.386; 95% CI: 1.253–9.15). Furthermore, higher air O_3_ levels have a negative functional effect on SD patients (*P* = 0.035). Existing studies have found air pollution to be causally linked to respiratory and allergic health problems [23, 24]. Over a decade ago, dermatologist Jean Krutmann began postulating how pollutants in the environment affect the skin. Evidence has shown that airborne pollutants harm the skin, and may even be deadly [13, 25, 26]. A recent epidemiological study has reported that indoor and outdoor air pollution also increase the risk of asthma, wheezing, rhinitis and eczema among pre-school children in China [27]. Due to the deleterious effects of airborne pollutants on both the skin and QoL, clinicians and seborrheic dermatitis patients may need to pay more attention to environmental air quality.

One limitation of this study should be mentioned. Owing to this non-random sampling design, there may have been selection bias. A larger sample size and random sampling are needed to collect more representative data for further research.

## Conclusion

Those who suffer from SD in China experience severe effects on all realms of daily life. We found that disease severity, dermatologic hospitalization and PM 10 level each had negative effects on patients’ QoL. These implications are alarming. Public health concerns for SD disease management, and its associated environmental factors, may see new emphasis in future SD mental health management.

## Supplementary information

**Additional file 1.** Environmental data in research sites between 2013 and 2015.

**Additional file 2.** Comparisons of the Skindex-29 cut-off score across subgroups.

## Data Availability

The final datasets are not publicly available. The corresponding author has access to the final dataset. However contractual agreements limit its disclosure. Investigators may be granted access upon reasonable request.

## Abbreviations

BMI: Body mass index
DLQI: Dermatology life quality index
PM: Particulate matter
QoL: Quality of life
SD: Seborrheic dermatitis
SD: Standard deviation
SF-36: Short form 36 questionnaire
