# Supplementary material for: Clinical characteristics and quality of life in seborrheic dermatitis patients: a cross-sectional study in China
Source: Health Qual Life Outcomes. 2020 Sep 16;18:308. doi: 10.1186/s12955-020-01558-y (PMC7493366; doi:10.1186/s12955-020-01558-y)
Supplement: Supplementary file 1 — Additional file 1. Environmental data in research sites between 2013 and 2015. [file 12955_2020_1558_MOESM1_ESM.docx]

Table 1 Environmental data in research sites between 2013 and 2015

| Environmental conditions | Year | City | | | | | |
| --- | --- | --- | --- | --- | --- | --- | --- |
|  |  | Guangzhou | Shanghai | Chengdu | Beijing | Urumchi | Harbin |
| SO_2_ (μg/m^3^) |  |  |  |  |  |  |  |
|  | 2013 | 20 | 24 | 31 | 26 | 29 | 44 |
|  | 2014 | 17 | 18 | 19 | 22 | 25 | 57 |
|  | 2015 | 13 | 17 | 14 | 14 | 15 | 40 |
| NO_2_ (μg/m^3^) |  |  |  |  |  |  |  |
|  | 2013 | 52 | 48 | 63 | 56 | 61 | 56 |
|  | 2014 | 48 | 45 | 59 | 57 | 56 | 52 |
|  | 2015 | 47 | 46 | 53 | 50 | 52 | 40 |
| CO(μg/m^3^) |  |  |  |  |  |  |  |
|  | 2013 | 1.5 | 1.6 | 2.6 | 3.4 | 5.9 | 2.2 |
|  | 2014 | 1.5 | 1.3 | 2 | 3.2 | 3.4 | 1.6 |
|  | 2015 | 1.4 | 1.5 | 2 | 3.6 | 3.6 | 1.8 |
| O_3_(μg/m^3^) |  |  |  |  |  |  |  |
|  | 2013 | 156 | 158 | 157 | 188 | 116 | 72 |
|  | 2014 | 165 | 149 | 147 | 200 | 109 | 111 |
|  | 2015 | 145 | 161 | 183 | 203 | 122 | 106 |
| PM2.5(μg/m^3^) |  |  |  |  |  |  |  |
|  | 2013 | 53 | 62 | 96 | 89 | 88 | 81 |
|  | 2014 | 49 | 52 | 77 | 86 | 61 | 72 |
|  | 2015 | 39 | 53 | 64 | 81 | 66 | 70 |
| PM10(μg/m^3^) |  |  |  |  |  |  |  |
|  | 2013 | 72 | 84 | 150 | 108 | 146 | 119 |
|  | 2014 | 67 | 71 | 123 | 116 | 146 | 111 |
|  | 2015 | 59 | 69 | 108 | 102 | 133 | 103 |
