# Supplementary material for: Clinical characteristics and quality of life in seborrheic dermatitis patients: a cross-sectional study in China
Source: Health Qual Life Outcomes. 2020 Sep 16;18:308. doi: 10.1186/s12955-020-01558-y (PMC7493366; doi:10.1186/s12955-020-01558-y)
Supplement: Supplementary file 2 — Additional file 2. Comparisons of the Skindex-29 cut-off score across subgroups. [file 12955_2020_1558_MOESM2_ESM.docx]

**Table 1 Comparison of the Skindex-29 emotion cut-off score across subgroups**

| Variable |  | <39 points  *n*(%) | ≥39 points  *n*(%) | Total | *χ^2^* | *P* value |
| --- | --- | --- | --- | --- | --- | --- |
| Sex |  |  |  |  |  |  |
|  | Male | 62(35) | 37(28.5) | 99 | 1.479 | 0.224 |
|  | Female | 115(65) | 93(71.5) | 208 |  |  |
| age |  |  |  |  |  |  |
|  | <24years | 55(30.9) | 47(36.2) | 102 | 0.937 | 0.333 |
|  | ≥24years | 123(69.1) | 83(63.8) | 206 |  |  |
| Duration |  |  |  |  |  |  |
|  | <3 years | 128(77.1) | 79(66.9) | 207 | 3.602 | 0.058 |
|  | ≥3 years | 38(22.9) | 39(33.1) | 77 |  |  |
| BMI |  |  |  |  |  |  |
|  | <25 | 150(86.7) | 120(95.2) | 270 | 6.061 | 0.014 |
|  | ≥25 | 23(13.3) | 6(4.8) | 29 |  |  |
| Relationship status |  |  |  |  |  |  |
|  | Married | 90(51.4) | 62(49.6) | 152 | 0.098 | 0.755 |
|  | Single | 85(48.6) | 63(50.4) | 148 |  |  |
| Highest level of education |  |  |  |  |  |  |
|  | High school education or less | 63(35.6) | 39(30.5) | 102 | 0.876 | 0.349 |
|  | College or above | 114(64.4) | 89(69.5) | 203 |  |  |
| Employment |  |  |  |  |  |  |
|  | Employed | 105(59.3) | 69(54.3) | 174 | 0.753 | 0.386 |
|  | Unemployed/student | 72(40.7) | 58(45.7) | 130 |  |  |
| Diet preference |  |  |  |  |  |  |
|  | No | 28(30.4) | 31(37.8) | 59 | 1.051 | 0.305 |
|  | Yes | 64(69.6) | 51(62.2) | 115 |  |  |
| Smoking |  |  |  |  |  |  |
|  | No | 121(68.8) | 74(57.8) | 195 | 3.855 | 0.050 |
|  | Yes | 55(31.3) | 54(42.2) | 109 |  |  |
| Alcohol consumption |  |  |  |  |  |  |
|  | No | 106(59.9) | 67(52.3) | 173 | 1.722 | 0.189 |
|  | Yes | 71(40.1) | 61(47.7) | 132 |  |  |
| Exercise |  |  |  |  |  |  |
|  | No | 81(45.8) | 52(41.9) | 133 | 0.433 | 0.510 |
|  | Yes | 96(54.2) | 72(58.1) | 168 |  |  |
| Hospitalized for skin disease over the previous year |  |  |  |  |  |  |
|  | No | 137(78.7) | 84(65.6) | 221 | 6.459 | 0.011 |
|  | Yes | 37(21.3) | 44(34.4) | 81 |  |  |
| Income |  |  |  |  |  |  |
|  | ≤4000yuan per month | 110(61.8) | 87(66.9) | 197 | 0.856 | 0.355 |
|  | >4000yuan per month | 68(38.2) | 43(33.1) | 111 |  |  |
| Medical insurance |  |  |  |  |  |  |
|  | No | 37(22.2) | 43(35) | 80 | 5.813 | 0.016 |
|  | Yes | 130(77.8) | 80(65) | 210 |  |  |
| SO_2_ level in the air |  |  |  |  |  |  |
|  | <20 μg‎/m^3^ | 78(43.8) | 55(42.3) | 133 | 1.685 | 0.431 |
|  | 20-40 μg‎/m^3^ | 51(28.7) | 31(23.8) | 82 |  |  |
|  | >40 μg‎/m^3^ | 49(27.5) | 44(33.8) | 93 |  |  |
| NO_2_ level in the air |  |  |  |  |  |  |
|  | ≤50 μg‎/m^3^ | 73(41) | 50(38.5) | 123 | 0.204 | 0.652 |
|  | >50 μg‎/m^3^ | 105(59) | 80(61.5) | 185 |  |  |
| CO level in the air |  |  |  |  |  |  |
|  | <2 μg‎/m^3^ | 112(62.9) | 86(66.2) | 198 | 0.384 | 0.825 |
|  | 2-4 μg‎/m^3^ | 55(30.9) | 36(27.7) | 91 |  |  |
|  | >4 μg‎/m^3^ | 11(6.2) | 8(6.2) | 19 |  |  |
| O_3_ level in the air |  |  |  |  |  |  |
|  | <100μg‎/m^3^ | 20(11.2) | 18(13.8) | 38 | 2.322 | 0.313 |
|  | 100-160μg‎/m^3^ | 81(45.5) | 48(36.9) | 129 |  |  |
|  | >160μg‎/m^3^ | 77(43.3) | 64(49.2) | 141 |  |  |
| PM2.5 level in the air |  |  |  |  |  |  |
|  | <60μg‎/m^3^ | 72(40.4) | 51(39.2) | 123 | 0.332 | 0.847 |
|  | 60-80μg‎/m^3^ | 84(47.2) | 60(46.2) | 144 |  |  |
|  | >80μg‎/m^3^ | 22(12.4) | 19(14.6) | 41 |  |  |
| PM10 level in the air |  |  |  |  |  |  |
|  | <70 μg‎/m^3^ | 65(36.5) | 47(36.2) | 112 | 7.362 | 0.025 |
|  | 70-120 μg‎/m^3^ | 71(39.9) | 67(51.5) | 138 |  |  |
|  | >120 μg‎/m^3^ | 42(23.6) | 16(12.3) | 58 |  |  |
| Disease severity |  |  |  |  |  |  |
|  | Slight or mild | 93(53.4) | 50(40) | 143 | 13.156 | 0.001 |
|  | Moderate | 76(43.7) | 59(47.2) | 135 |  |  |
|  | Severe | 5(2.9) | 16(12.8) | 21 |  |  |

The Skindex-29 cut-off scores evaluated severely emotional QoL impairment is ≥39 points.

**Table 2 Comparison of the Skindex-29 symptom cut-off score across subgroups**

| Variable |  | <52 points  *n*(%) | ≥52 points  *n*(%) | Total | *χ^2^* | *P* value |
| --- | --- | --- | --- | --- | --- | --- |
| Sex |  |  |  |  |  |  |
|  | Male | 77(33.2) | 22(29.3) | 99 | 0.386 | 0.535 |
|  | Female | 155(66.8) | 53(70.7) | 208 |  |  |
| age |  |  |  |  |  |  |
|  | <24years | 83(35.6) | 19(25.3) | 102 | 2.712 | 0.100 |
|  | ≥24years | 150(64.4) | 56(74.7) | 206 |  |  |
| Duration |  |  |  |  |  |  |
|  | <3 years | 161(75.2) | 46(65.7) | 207 | 2.419 | 0.120 |
|  | ≥3 years | 53(24.8) | 24(34.3) | 77 |  |  |
| BMI |  |  |  |  |  |  |
|  | <25 | 203(89.8) | 67(91.8) | 270 | 0.241 | 0.623 |
|  | ≥25 | 23(10.2) | 6(8.2) | 29 |  |  |
| Relationship status |  |  |  |  |  |  |
|  | Married | 110(48.2) | 42(58.3) | 152 | 2.228 | 0.136 |
|  | Single | 118(51.8) | 30(41.7) | 148 |  |  |
| Highest level of education |  |  |  |  |  |  |
|  | High school education or less | 81(35.1) | 21(28.4) | 102 | 1.126 | 0.289 |
|  | College or above | 150(64.9) | 53(71.6) | 203 |  |  |
| Employment |  |  |  |  |  |  |
|  | Employed | 131(57) | 43(58.1) | 174 | 0.030 | 0.862 |
|  | Unemployed/student | 99(43) | 31(41.9) | 130 |  |  |
| Diet preference |  |  |  |  |  |  |
|  | No | 43(32.3) | 16(39) | 59 | 0.627 | 0.429 |
|  | Yes | 90(67.7) | 25(61) | 115 |  |  |
| Smoking |  |  |  |  |  |  |
|  | No | 150(65.2) | 45(60.8) | 195 | 0.473 | 0.492 |
|  | Yes | 80(34.8) | 29(39.2) | 109 |  |  |
| Alcohol consumption |  |  |  |  |  |  |
|  | No | 133(57.6) | 40(54.1) | 173 | 0.283 | 0.595 |
|  | Yes | 98(42.4) | 34(45.9) | 132 |  |  |
| Exercise |  |  |  |  |  |  |
|  | No | 101(44.1) | 32(44.4) | 133 | 0.003 | 0.960 |
|  | Yes | 128(55.9) | 40(55.6) | 168 |  |  |
| Hospitalized for skin disease over the previous year |  |  |  |  |  |  |
|  | No | 170(74.6) | 51(68.9) | 221 | 0.906 | 0.341 |
|  | Yes | 58(25.4) | 23(31.1) | 81 |  |  |
| Income |  |  |  |  |  |  |
|  | ≤4000yuan per month | 148(63.5) | 49(65.3) | 197 | 0.081 | 0.776 |
|  | >4000yuan per month | 85(36.5) | 26(34.7) | 111 |  |  |
| Medical insurance |  |  |  |  |  |  |
|  | No | 55(25.2) | 25(34.7) | 80 | 2.442 | 0.118 |
|  | Yes | 163(74.8) | 47(65.3) | 210 |  |  |
| SO_2_ level in the air |  |  |  |  |  |  |
|  | <20 μg‎/m^3^ | 102(43.8) | 31(41.3) | 133 | 0.464 | 0.793 |
|  | 20-40 μg‎/m^3^ | 63(27) | 19(25.3) | 82 |  |  |
|  | >40 μg‎/m^3^ | 68(29.2) | 25(33.3) | 93 |  |  |
| NO_2_ level in the air |  |  |  |  |  |  |
|  | ≤50 μg‎/m^3^ | 96(41.2) | 27(36) | 123 | 0.640 | 0.424 |
|  | >50 μg‎/m^3^ | 137(58.8) | 48(64) | 185 |  |  |
| CO level in the air |  |  |  |  |  |  |
|  | <2 μg‎/m^3^ | 150(64.4) | 48(64) | 198 | 0.156 | 0.925 |
|  | 2-4 μg‎/m^3^ | 68(29.2) | 23(30.7) | 91 |  |  |
|  | >4 μg‎/m^3^ | 15(6.4) | 4(5.3) | 19 |  |  |
| O_3_ level in the air |  |  |  |  |  |  |
|  | <100μg‎/m^3^ | 30(12.9) | 8(10.7) | 38 | 0.582 | 0.748 |
|  | 100-160μg‎/m^3^ | 99(42.5) | 30(40) | 129 |  |  |
|  | >160μg‎/m^3^ | 104(44.6) | 37(49.3) | 141 |  |  |
| PM2.5 level in the air |  |  |  |  |  |  |
|  | <60μg‎/m^3^ | 96(41.2) | 27(36) | 123 | 0.956 | 0.620 |
|  | 60-80μg‎/m^3^ | 108(46.4) | 36(48) | 144 |  |  |
|  | >80μg‎/m^3^ | 29(12.4) | 12(16) | 41 |  |  |
| PM10 level in the air |  |  |  |  |  |  |
|  | <70 μg‎/m^3^ | 86(36.9) | 26(34.7) | 112 | 0.954 | 0.621 |
|  | 70-120 μg‎/m^3^ | 101(43.3) | 37(49.3) | 138 |  |  |
|  | >120 μg‎/m^3^ | 46(19.7) | 12(16) | 58 |  |  |
| Disease severity |  |  |  |  |  |  |
|  | Slight or mild | 119(52.9) | 24(32.4) | 143 | 20.951 | 0.00 |
|  | Moderate | 98(43.6) | 37(50) | 135 |  |  |
|  | Severe | 8(3.6) | 13(17.6) | 21 |  |  |

The Skindex-29 cut-off scores evaluated severely symptomatic QoL impairment is ≥52 points.

**Table 3 Comparison of the Skindex-29 functioning cut-off score across subgroups**

| Variable |  | <37 points  n(%) | ≥37 points  n(%) | Total | *χ^2^* | *P* value |
| --- | --- | --- | --- | --- | --- | --- |
| Sex |  |  |  |  |  |  |
|  | Male | 79(32.8) | 20(30.3) | 99 | 0.146 | 0.703 |
|  | Female | 162(67.2) | 46(69.7) | 208 |  |  |
| age |  |  |  |  |  |  |
|  | <24years | 80(33.1) | 22(33.3) | 102 | 0.002 | 0.966 |
|  | ≥24years | 162(66.9) | 44(66.7) | 206 |  |  |
| Duration |  |  |  |  |  |  |
|  | <3 years | 165(74.3) | 42(67.7) | 207 | 1.063 | 0.303 |
|  | ≥3 years | 57(25.7) | 20(32.3) | 77 |  |  |
| BMI |  |  |  |  |  |  |
|  | <25 | 210(89) | 60(95.2) | 270 | 2.221 | 0.136 |
|  | ≥25 | 26(11) | 3(4.8) | 29 |  |  |
| Relationship status |  |  |  |  |  |  |
|  | Married | 118(49.8) | 34(54) | 152 | 0.348 | 0.555 |
|  | Single | 119(50.2) | 29(46) | 148 |  |  |
| Highest level of education |  |  |  |  |  |  |
|  | High school education or less | 80(33.3) | 22(33.8) | 102 | 0.006 | 0.938 |
|  | College or above | 160(66.7) | 43(66.2) | 203 |  |  |
| Employment |  |  |  |  |  |  |
|  | Employed | 136(56.7) | 38(59.4) | 174 | 0.151 | 0.697 |
|  | Unemployed/student | 104(43.3) | 26(40.6) | 130 |  |  |
| Diet preference |  |  |  |  |  |  |
|  | No | 40(30.3) | 19(45.2) | 59 | 3.171 | 0.075 |
|  | Yes | 92(69.7) | 23(54.8) | 115 |  |  |
| Smoking |  |  |  |  |  |  |
|  | No | 159(66.5) | 36(55.4) | 195 | 2.759 | 0.097 |
|  | Yes | 80(33.5) | 29(44.6) | 109 |  |  |
| Alcohol consumption |  |  |  |  |  |  |
|  | No | 141(58.8) | 32(49.2) | 173 | 1.888 | 0.169 |
|  | Yes | 99(41.3) | 33(50.8) | 132 |  |  |
| Drinking |  |  |  |  |  |  |
|  | No | 106(44.5) | 27(42.9) | 133 | 0.057 | 0.811 |
|  | Yes | 132(55.5) | 36(57.1) | 168 |  |  |
| Hospitalized for skin disease over the previous year |  |  |  |  |  |  |
|  | No | 183(76.9) | 38(59.4) | 221 | 7.884 | 0.005 |
|  | Yes | 55(23.1) | 26(40.6) | 81 |  |  |
| Income |  |  |  |  |  |  |
|  | ≤4000yuan per month | 157(64.9) | 40(60.6) | 197 | 0.410 | 0.522 |
|  | >4000yuan per month | 85(35.1) | 26(39.4) | 111 |  |  |
| Medical insurance |  |  |  |  |  |  |
|  | No | 58(25.6) | 22(34.9) | 80 | 2.167 | 0.141 |
|  | Yes | 169(74.4) | 41(65.1) | 210 |  |  |
| SO_2_ level in the air |  |  |  |  |  |  |
|  | <20 μg‎/m^3^ | 103(42.6) | 30(45.5) | 133 | 1.513 | 0.469 |
|  | 20-40 μg‎/m^3^ | 62(25.6) | 20(30.3) | 82 |  |  |
|  | >40 μg‎/m^3^ | 77(31.8) | 16(24.2) | 93 |  |  |
| NO_2_ level in the air |  |  |  |  |  |  |
|  | ≤50 μg‎/m^3^ | 96(39.7) | 27(40.9) | 123 | 0.033 | 0.55 |
|  | >50 μg‎/m^3^ | 146(60.3) | 39(59.1) | 185 |  |  |
| CO level in the air |  |  |  |  |  |  |
|  | <2 μg‎/m^3^ | 158(65.3) | 40(60.6) | 198 | 1.335 | 0.513 |
|  | 2-4 μg‎/m^3^ | 68(28.1) | 23(34.8) | 91 |  |  |
|  | >4 μg‎/m^3^ | 16(6.6) | 3(4.5) | 19 |  |  |
| O_3_ level in the air |  |  |  |  |  |  |
|  | <100μg‎/m^3^ | 30(12.4) | 8(12.1) | 38 | 6.694 | 0.035 |
|  | 100-160μg‎/m^3^ | 110(45.5) | 19(28.8) | 129 |  |  |
|  | >160μg‎/m^3^ | 102(42.1) | 39(59.1) | 141 |  |  |
| PM2.5 level in the air |  |  |  |  |  |  |
|  | <60μg‎/m^3^ | 94(38.8) | 29(43.9) | 123 | 4.849 | 0.089 |
|  | 60-80μg‎/m^3^ | 120(49.6) | 24(36.4) | 144 |  |  |
|  | >80μg‎/m^3^ | 28(11.6) | 13(19.7) | 41 |  |  |
| PM2.5 level in the air |  |  |  |  |  |  |
|  | <70 μg‎/m^3^ | 85(35.1) | 27(40.9) | 112 | 1.689 | 0.430 |
|  | 70-120 μg‎/m^3^ | 108(44.6) | 30(45.5) | 138 |  |  |
|  | >120 μg‎/m^3^ | 49(20.2) | 9(13.6) | 58 |  |  |
| Disease severity |  |  |  |  |  |  |
|  | Slight or mild | 123(52.6) | 20(30.8) | 143 | 21.105 | 0.000 |
|  | Moderate | 102(43.6) | 33(50.8) | 135 |  |  |
|  | Severe | 9(3.8) | 12(18.5) | 21 |  |  |

The Skindex-29 cut-off scores evaluated severely functional QoL impairment is ≥37 points.

**Table 4 Comparison of the Skindex-29 cut-off overall score across subgroups**

| Variable |  | <44 points  *n*(%) | ≥44 points  *n*(%) | Total | *χ^2^* | *P* value |
| --- | --- | --- | --- | --- | --- | --- |
| Sex |  |  |  |  |  |  |
|  | Male | 76(34.4) | 23(26.7) | 99 | 1.656 | 0.198 |
|  | Female | 145(65.6) | 63(73.3) | 208 |  |  |
| age |  |  |  |  |  |  |
|  | <24years | 71(32) | 31(36) | 102 | 0.462 | 0.497 |
|  | ≥24years | 151(68) | 55(64) | 206 |  |  |
| Duration |  |  |  |  |  |  |
|  | <3 years | 154(75.5) | 53(66.3) | 207 | 2.483 | 0.115 |
|  | ≥3 years | 50(24.5) | 27(33.8) | 77 |  |  |
| BMI |  |  |  |  |  |  |
|  | <25 | 190(88.4) | 80(95.2) | 270 | 3.251 | 0.071 |
|  | ≥25 | 25(11.6) | 4(4.8) | 29 |  |  |
| Relationship status |  |  |  |  |  |  |
|  | Married | 111(50.9) | 41(50) | 152 | 0.02 | 0.887 |
|  | Single | 107(49.1) | 41(50) | 148 |  |  |
| Highest level of education |  |  |  |  |  |  |
|  | High school education or less | 75(34.1) | 27(31.8) | 102 | 0.149 | 0.699 |
|  | College or above | 145(65.9) | 58(68.2) | 203 |  |  |
| Employment |  |  |  |  |  |  |
|  | Employed | 127(57.7) | 47(56) | 174 | 0.078 | 0.780 |
|  | Unemployed/student | 93(42.3) | 37(44) | 130 |  |  |
| Diet preference |  |  |  |  |  |  |
|  | No | 36(30) | 23(42.6) | 59 | 2.635 | 0.105 |
|  | Yes | 84(70) | 31(57.4) | 115 |  |  |
| Smoking |  |  |  |  |  |  |
|  | No | 149(68) | 46(54.1) | 195 | 5.158 | 0.023 |
|  | Yes | 70(32) | 39(45.9) | 109 |  |  |
| Alcohol consumption |  |  |  |  |  |  |
|  | No | 133(60.5) | 40(47.1) | 173 | 4.482 | 0.034 |
|  | Yes | 87(39.5) | 45(52.9) | 132 |  |  |
| Exercise |  |  |  |  |  |  |
|  | No | 98(45) | 35(42.2) | 133 | 0.189 | 0.664 |
|  | Yes | 120(55) | 48(57.8) | 168 |  |  |
| Hospitalized for skin disease over the previous year |  |  |  |  |  |  |
|  | No | 170(78) | 51(60.7) | 221 | 9.211 | 0.002 |
|  | Yes | 48(22) | 33(39.3) | 81 |  |  |
| Income |  |  |  |  |  |  |
|  | ≤4000yuan per month | 141(63.5) | 56(65.1) | 197 | 0.069 | 0.793 |
|  | >4000yuan per month | 81(36.5) | 30(34.9) | 111 |  |  |
| Medical insurance |  |  |  |  |  |  |
|  | No | 50(24.3) | 30(35.7) | 80 | 3.911 | 0.048 |
|  | Yes | 156(75.7) | 54(64.3) | 210 |  |  |
| SO_2_ level in air |  |  |  |  |  |  |
|  | <20 μg‎/m^3^ | 95(42.8) | 38(44.2) | 133 | 0.077 | 0.962 |
|  | 20-40 μg‎/m^3^ | 60(27) | 22(25.6) | 82 |  |  |
|  | >40 μg‎/m^3^ | 67(30.2) | 26(30.2) | 93 |  |  |
| NO_2_ level in air |  |  |  |  |  |  |
|  | ≤50 μg‎/m^3^ | 88(39.6) | 35(40.7) | 123 | 0.029 | 0.865 |
|  | >50 μg‎/m^3^ | 134(60.4) | 51(59.3) | 185 |  |  |
| CO level in air |  |  |  |  |  |  |
|  | <2 μg‎/m^3^ | 141(63.5) | 57(66.3) | 198 | 1.482 | 0.477 |
|  | 2-4 μg‎/m^3^ | 65(29.3) | 26(30.2) | 91 |  |  |
|  | >4 μg‎/m^3^ | 16(7.2) | 3(3.5) | 19 |  |  |
| O_3_ level in air |  |  |  |  |  |  |
|  | <100μg‎/m^3^ | 29(13.1) | 9(10.5) | 38 | 4.861 | 0.088 |
|  | 100-160μg‎/m^3^ | 100(45) | 29(33.7) | 129 |  |  |
|  | >160μg‎/m^3^ | 93(41.9) | 48(55.8) | 141 |  |  |
| PM2.5 level in air |  |  |  |  |  |  |
|  | <60μg‎/m^3^ | 86(38.7) | 37(43) | 123 | 2.010 | 0.366 |
|  | 60-80μg‎/m^3^ | 109(49.1) | 35(40.7) | 144 |  |  |
|  | >80μg‎/m^3^ | 27(12.2) | 14(16.3) | 41 |  |  |
| PM10 level in air |  |  |  |  |  |  |
|  | <70 μg‎/m^3^ | 78(35.1) | 34(39.5) | 112 | 5.483 | 0.064 |
|  | 70-120 μg‎/m^3^ | 95(42.8) | 43(50) | 138 |  |  |
|  | >120 μg‎/m^3^ | 49(22.1) | 9(10.5) | 58 |  |  |
| Disease severity |  |  |  |  |  |  |
|  | Slight or mild | 116(54) | 27(32.1) | 143 | 22.419 | 0.000 |
|  | Moderate | 92(42.8) | 43(51.2) | 135 |  |  |
|  | Severe | 7(3.3) | 14(16.7) | 21 |  |  |

The Skindex-29 cut-off overall scores evaluated severely QoL impairment is ≥44 points.
